# Fatal Systemic Vasoconstriction in a Case of Metastatic Small-Intestinal NET

**DOI:** 10.1155/2017/9810194

**Published:** 2017-07-18

**Authors:** Jochen Stenzel, Sebastian Noe, Konstantin Holzapfel, Franziska Erlmeier, Florian Eyer

**Affiliations:** ^1^Department of Clinical Toxicology, Klinikum rechts der Isar, Technical University of Munich, Ismaningerstrasse 22, 81675 Munich, Germany; ^2^Department of Internal Medicine II (Gastroenterology), Klinikum rechts der Isar, Technical University of Munich, Ismaningerstrasse 22, 81675 Munich, Germany; ^3^Department of Diagnostic and Interventional Radiology, Klinikum rechts der Isar, Technical University of Munich, Ismaningerstrasse 22, 81675 Munich, Germany; ^4^Institute of Pathology, Klinikum rechts der Isar, Technical University of Munich, Ismaningerstrasse 22, 81675 Munich, Germany

## Abstract

An increased release of serotonin secreted by ileal NETs is thought to be the major factor causing the carcinoid syndrome. However, in acutely arising carcinoid crisis also other vasoactive factors may lead to hazardous fluctuations in blood pressure and bronchial constriction. In rare cases, systemic vasoconstriction can be observed, probably caused by catecholamines or similar acting substances. Here, we report a fatal case of fulminant systemic vasoconstriction possibly caused by catecholamines in a patient with metastasized ileal NET. The vasospasm was detected by CT-angiography, and hemodynamic monitoring revealed a high systemic vascular resistance. Epinephrine, norepinephrine, and chromogranin A levels in plasma were elevated as was the urinary 5-hydroxyindoleacetic acid (5-HIAA). The cause of death was heart failure due to severe circulatory insufficiency. The progression of the tumor disease was confirmed by autopsy.

## 1. Introduction

Neuroendocrine tumors (NET) of the gastroenteropancreatic system are low-grade malignant neoplasms derived from local neuroendocrine cells [1].

Their clinical presentation largely depends on primary tumor site and whether or not significant amounts of hormones are produced and secreted. The predominantly secreted hormonally active substances, such as polypeptides, biogenic amines, and prostaglandins, vary, depending on tumor localization [2–5]. NETs of the ileum primarily release serotonin (5-hydroxytryptophan (5-HT)) and metastasize to the lymph nodes and liver resulting in a reduced presystemic metabolism of hormonal active substances by the liver [2, 6, 7].

An excessive release of these active substances caused by different triggers like surgery/anaesthesia, interventional diagnostic and therapy, radionuclide therapy, medication, and examination can lead to carcinoid crisis characterized by dramatic blood pressure fluctuation, arrhythmias, bronchospasm, and mental disturbances [8–13]. Commonly, hypotension is the expected hemodynamic change; however a small group of carcinoid patients experiences hypertension during carcinoid crisis [14, 15]. The occurrence of localized or generalized vasospasms associated with NETs has been described in case reports only [16–18]; see also Table 2.

Here, we report a fatal case of a metastasized syndromic NET of the terminal ileum with systemic vasospasm resulting in secondary low-output syndrome and consecutive multiorgan failure. CT-angiography, advanced hemodynamic monitoring, and determination of catecholamines, chromogranin A in plasma, and 5-hydroxyindoleacetic acid (5-HIAA) in urine were performed. The cause of death was determined post-mortem by autopsy.

## 2. Case Report

In 2011, a liver biopsy of hepatic lesions of a 51-year-old female revealed liver metastases from a well-differentiated small intestine NET (G1, Ki 67 max. 3%).

**Figure 1:**
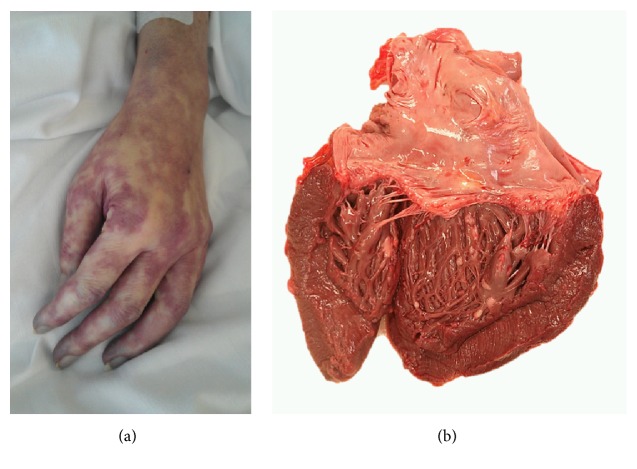
(a) Skin with regional vasospasm; (b) opened heart, showing multiple small white metastases of a midgut NET.

**Figure 2:**
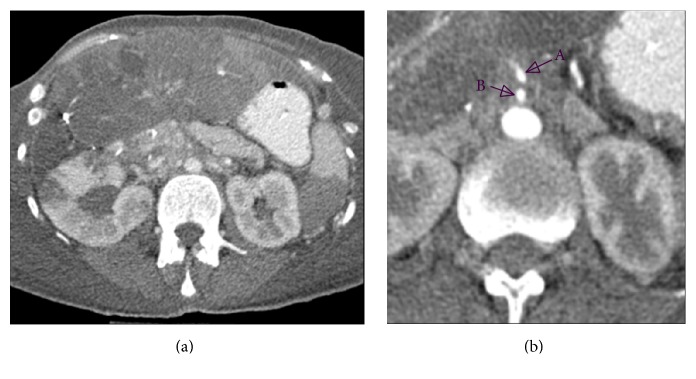
Abdominal CT-scan. (a) Centralized perfusion with constricted peripheral intestinal arteries. (b) Reduced diameters of abdominal arteries. (A) Truncus coeliacus: 3 mm. (B) A. mesenterica sup.: 3 mm; aorta 15 mm.

**Figure 3:**
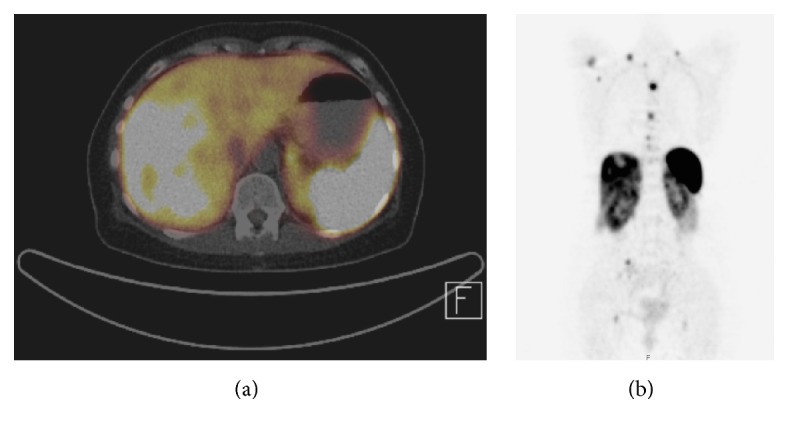
Somatostatin receptor imaging with CT-scan (PET-CT) (a) showing extensive liver metastasis in the right liver lobe in 12/2011 and (b) additionally showing hydronephrosis.

**Figure 4:**
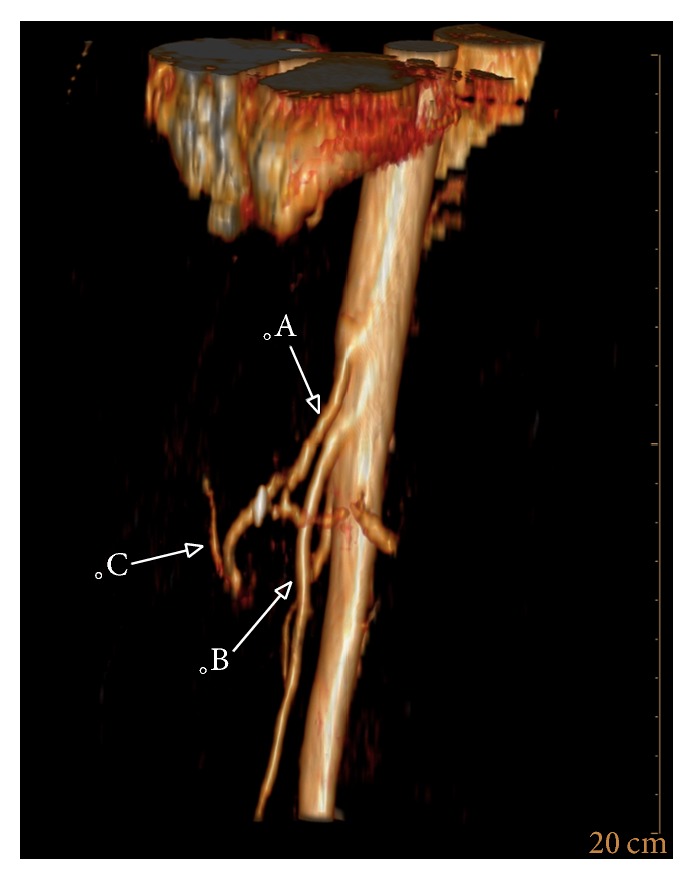
3D reconstruction of the abdominal arteries showing the rarefication of the peripheral hepatic arteries. (A) Truncus coeliacus. (B) A. mesenterica sup. (C) Branch of the constricted hepatic artery.

A DOTA-TOC-PET/CT demonstrated metastatic disease with a suspected ileal primary; see Figure 3. After hemihepatectomy and resection of the primary tumor in the ileum, histopathology of the terminal ileum showed a serotonin-producing, well-differentiated NET. Flush-symptoms were controlled under treatment with somatostatin analogues. With reappearance of the symptoms after almost one year, another DOTA-NOC-PET-CT showed distinct ubiquitary tumor progress. It was therefore decided to perform a peptide radioreceptor radionuclide therapy (PRRT) in 2013. About 4 days after the beginning of the PRRT, her general condition deteriorated dramatically. The patient was transferred to our ICU due to impaired consciousness, progressive delirium, and increase of inflammatory parameters (CRP 16 mg/dL, WBC 23.6 G/L) for suspected urosepsis originating from a chronic second grade hydronephrosis. After admission the patient showed hypertensive blood pressure values measured via a femoral artery catheter in contrast to hypotensive noninvasive blood pressure determinations. Blood culture revealed Klebsiella pneumoniae sensitive to empiric antibiotics. However, despite decreasing inflammation parameters, the patient's condition deteriorated with progression of liver and renal failure, delirium, and bloody diarrhoea. A pattern reminiscent of dermal livedo reticularis appeared (Figure 1) and a thoracic-abdominal contrast CT-scan revealed a massive and generalized arterial vasoconstriction with infarction of the kidneys, liver, and spleen (Figures 2 and 4). Massively elevated systemic vascular resistance index (SVRI) and a reduced cardiac index (CI) were measured using an advanced hemodynamic monitoring (PICCO®, Pulsion Medical Systems SE); see Table 1. Markers of myocardial necrosis along with NT-proBNP (max. 1287 pg/ml) steadily increased because of progressive cardiac failure. In contrast, an echocardiography performed in 2013 showed a normal left/right ventricular function and a first grade mitral and tricuspid regurgitation. Peripheral hydropic decompensation with extensive bilateral pleural effusions, oliguria, absence of hypotension, arterial hypoxemia, and ultimately the end stage condition of the patient thwarted aggressive volume therapy.

An excess of vasoactive amines secreted by the NET was now regarded as the most likely reason for clinical deterioration. Massively elevated chromogranin A in plasma and, notably, elevated catecholamine plasma levels (in pg/ml) supported this hypothesis: epinephrine 2318.3 (50–100 pg/ml), norepinephrine 1339.3 (<400 pg/mL), metanephrine 170.2 (<90 pg/mL), normetanephrine 349,5  (<180 pg/mL), and chromogranin A (in ng/ml, <84.7 ng/ml) 896 (11/2011), 717 (06/2012), 1.120 (01/2013), and 16.310 6 days before and 33.580 6 days after ICU admission. In contrast, the 5-HIAA levels in urine were elevated but remained stable: 66.2 mg/12 h on day 6 after admission and 153 mg/24 h 452 days before admission (1–10 mg/24 h); see Table 3.

Octreotide dose was increased and administered intravenously combined with orally administered cyproheptadine. Treatment attempts with nimodipine, phentolamine, and dobutamine aiming at reducing SVRI and cardiac afterload and thereby improving the CI were largely ineffective. Respiratory insufficiency worsened but intubation and mandatory ventilation were not performed in agreement with the family due to the underlying progressive malignancy. The patient finally died 7 days after ICU admission.

At autopsy the affected organs included the heart (disseminated pericardial, intramural, and endocardial metastases with involvement of the papillary muscles; histologically, approximately 60% vital tumor mass persisted after therapy; angioinvasion; no endocardial fibrosis), lungs (disseminated metastases and perivascular), stomach, pancreas, soft tissues, thyroid gland, ovaries, and bones. Histologically, a partial treatment response was found. The final TNM UICC classification was aT3 (m), aN1 (2/2), aM1 (PUL, HEP, PER, OTH, OSS, and LYM), aL1, aV1, and aPn1; grading G1. The cause of death was determined as heart failure due to severe circulatory insufficiency.

## 3. Discussion

The dramatic deterioration in our patient was caused by a progressive cardiac failure with increased vascular resistance and low cardiac index leading to a secondary low-output syndrome.

The massive increase of chromogranin A suggested a carcinoid crisis, although presenting with an atypical systemic vasoconstriction instead of more commonly found vasodilatation and might have possibly been triggered by the preceding PRRT.

Excluding cardiac valvular lesions, the main symptoms of the typical carcinoid syndrome with cutaneous flushing, venous telangiectasia, diarrhoea, and bronchospasm were present for about 2 years in our patient. Functional midgut NETs mainly secret serotonin leading to the typical carcinoid syndrome. Depending on the location of the NET (foregut, hindgut, and pulmonary) other hormones can predominate in systemic circulation resulting in an atypical carcinoid syndrome with variant clinical symptoms [22]. Carcinoid crisis is the life-threatening form of the carcinoid syndrome typically accompanied by severe hypotension. It can be triggered during tumor surgery by manipulation at tumor and metastatic sites or anaesthesia and has, yet more rarely, been described after chemotherapy, hepatic arterial embolization, or radionuclide therapy [23–25]. In a minority of patients, hypertension emerges during carcinoid crisis. This phenomenon was sparsely reported so far. In 1968 Rosenberg published a case report with additional 22 cases of previous literature with hypertensive carcinoid crisis [14]. Warner et al. reported another 2 cases and a review of literature. Based on these observations, (pre)treatment with octreotide is recommended for therapeutic interventions in functional midgut NET. Although some drugs have been successfully used in certain cases, for example, cyproheptadine, ketanserin, 5-HT receptor antagonists, corticosteroids, and H1- and H2-receptor antagonists [15], somatostatin analogues are considered most effective and are recommended as first-line therapy [8, 9, 26].

Still, there are differing opinions regarding octreotide pretreatment against carcinoid crisis before invasive procedures. A retrospective study evaluating the perioperative high-dose octreotide infusion showed a minimized incidence of carcinoid crisis [26]. In contrast, in a meta-analysis, the efficacy of somatostatin analogues to prevent carcinoid crisis could not be confirmed [8].

Pharmacological interventions to overcome the systemic vasoconstriction ultimately failed, despite the use of potent vasodilators. Treatment with nimodipine applied in the early phase of the ICU course seemed to be more effective compared to phentolamine. Intravenous octreotide applied in a dose of 500 *μ*g/24 h continuously iv was also ineffective (recommended dose for carcinoid crisis 50–600 *μ*g/day iv). There are reports of the successful administration of 150 *μ*g/h iv continuously over several days or of a bolus dose of up to 1,5 mg iv [9].

Both sepsis and previous PRRT might have served as a trigger but despite effective sepsis therapy the patient's condition worsened. Furthermore, a high SVRI along with a low CI strongly indicates the development of a cardiogenic shock; see Table 1 [27].

The renal function was normal before PRRT with a creatinine of 0.8 mg/dL and slightly impaired after ICU admission (creatinine 1.6 mg/dL, maximum 2.0 mg/dl). In the long term, PRRT can worsen renal function [28]. We postulate that the acute hemodynamic changes in short term are significantly more likely the cause of the acute renal failure in our patient.

Myocardial tumor infiltration may have additionally contributed to the development of cardiac failure, but the cardiac metastases with a maximum diameter of only 1.5 cm were not pronounced enough to explain the finding sufficiently. Cardiac metastases of a midgut NET are rare but may occur [29]. Interestingly, none of the diagnostic imaging—including echocardiography, MRT, and PET-CT performed one and two months previously—showed any signs of cardiac tumor manifestation. Markers of myocardial necrosis along with NT-proBNP steadily increased during the clinical course of our patient and indicated progressive cardiac failure. However, the pathomechanism compared to coronary heart disease may be reversed in case of primarily exceeding humoral vasoconstrictive factors. The development of heart failure occurred secondary to the pronounced increased vascular resistance with the need of an augmented heart work against a high afterload.

In our case, only a minimal increase of 5-HIAA levels in urine was observed and this might indicate a less pronounced serotonin excess. However, the value was derived from a 12-hour period urine collection and an oliguric kidney injury was present; see Table 3.

It has suggested that serotonin may lead to ergotism, a similar condition of systemic vasoconstriction [19]. Serotonin is able to release noradrenaline in several adrenergic innervated tissues [30] and can amplify the effect of epinephrine, norepinephrine, and angiotensin II [31].

Midgut NETs are able to excrete catecholamines [32]. A single determination of serum catecholamines in our patient showed remarkably elevated catecholamine levels in blood. Epinephrine levels were twice as high as those, for example, observed in stress cardiomyopathy [33].

By immunohistochemically staining of catecholamine-synthesizing enzymes it has been shown that the enzyme pathway for catecholamines in midgut NETs is similar to that in pheochromocytomas [34].

## 4. Conclusion

Serotonin is mainly assumed to be responsible for the carcinoid crisis. Based on the clinical presentation of our patient with a concomitant excess of catecholamines, we suggest that catecholamines at last contributed to the occurrence of the observed generalized systemic vasoconstriction, ultimately leading to acute heart failure.

## Figures and Tables

**Table 1:** Hemodynamic parameters.

Hours after admission to ICU^**∗**^	99,4	100,9	104,5	115,9	117	118	119,4	120	138,5	139,2	141,1	141.7
CI^*∗*^	2.13	2.09	2.14	1.36	1.36	1.1	1.8		1.73	1.6	1.18	2.18
SVRI^*∗∗*^	4247	3523	1526	3092	2200	7500	2916	3500	3034	3875	2857	2505

^*∗*^Admission hour *h* = 0 (reference values: ^*∗*^3.0–5.0 l/min/m^2^; ^*∗∗*^1700–2400 dyn*∗*s*∗*cm^−5^ *∗*m^2^).

**Table 2:** Similar case reports in literature.

Clinical presentation	Site of tumor	Publication
Coronary vasospasm	Liver metastases of unknown origin	[16, 17]
	Carcinoid tumor, originated from ileum	[18]
Systemic vasoconstriction	Pulmonary NET	[19]
Catecholamine producing	Ileal NET	[20]
	GEP NET with diffuse liver metastases	[21]

**Table 3:** Hormone levels in blood and urine.

	Days before and after admission (admission = *d* 0)							
	*d* −476	*d* −452	*d* −364	*d* −266	*d* −58	*d* −56	*d* −6	*d* 6
Chromogranin A (plasma^*∗*,*∗∗*^)	896^*∗∗*^		748^*∗∗*^	717^*∗∗*^	1120^*∗*^		16310^*∗*^	33580^*∗*^
5-HIES (urine/24 h, 1–10 mg/24 h)		153				121		132,4^#^

^*∗∗*^CIS Bio Radioimmunoassay (normal levels < 84.7 ng/ml). ^*∗*^Brahms Immunoassay/Thermofisher (normal levels < 110 ng/ml). ^#^Urine was collected over a 12-hour period.

## Abbreviations

CI: Cardiac index
DOTA-NOC: [^68^Ga-DOTA^0^-1NaI^3^]octreotide
DOTA-TOC: [^68^Ga-DOTA^0^-Phe^1^-Tyr^3^]octreotide
5-HIAA: 5-Hydroxyindoleacetic acid
5-HT: 5-Hydroxytryptophan
NET: Neuroendocrine tumor
NT-proBNP: N-terminal of the prohormone brain natriuretic peptide
PRRT: Peptide radioreceptor radionuclide therapy
SVRI: Systemic vascular resistance index.
