# Corrigendum to “Fatal Systemic Vasoconstriction in a Case of Metastatic Small-Intestinal NET”

**DOI:** 10.1155/2017/8694296

**Published:** 2017-12-03

**Authors:** Jochen Stenzel, Sebastian Noe, Konstantin Holzapfel, Franziska Erlmeier, Florian Eyer

**Affiliations:** ^1^Department of Clinical Toxicology, Klinikum rechts der Isar, Technical University of Munich, Ismaningerstrasse 22, 81675 Munich, Germany; ^2^Department of Internal Medicine II (Gastroenterology), Klinikum rechts der Isar, Technical University of Munich, Ismaningerstrasse 22, 81675 Munich, Germany; ^3^Department of Diagnostic and Interventional Radiology, Klinikum rechts der Isar, Technical University of Munich, Ismaningerstrasse 22, 81675 Munich, Germany; ^4^Institute of Pathology, Klinikum rechts der Isar, Technical University of Munich, Ismaningerstrasse 22, 81675 Munich, Germany

In the article titled “Fatal Systemic Vasoconstriction in a Case of Metastatic Small-Intestinal NET” [1], the first and last names of all the authors were reversed. The revised authors' list is shown above and updated in place.
